# Relevance of functional studies for assessing an antisense oligonucleotide-mediated exon skipping therapeutic strategy for mucolipidosis type II

**DOI:** 10.1186/s13023-026-04430-2

**Published:** 2026-06-17

**Authors:** Mariana Gonçalves, Marisa Encarnação, Luciana Moreira, Paulo Gaspar, Ana Joana Duarte, Maria Francisca Coutinho, Juliana Inês Santos, Maria João Prata, Maryam Omidi, Naemi Euchner, Sandra Pohl, Frederico Silva, Paula Oliveira, Liliana Matos, Sandra Alves

**Affiliations:** 1 Research and Development Unit, Department of Human Genetics, National Health Institute Dr. Ricardo Jorge, Porto, Portugal; 2 Center for the Study of Animal Science, CECA, Medical and Biomedical Sciences School, Fernando Pessoa University, Gondomar, Porto, Portugal; 3 Associate Laboratory for Animal and Veterinary Sciences, Faculty of Veterinary Medicine, University of Lisbon, Lisbon, Portugal; 4 Center for the Research and Technology of Agro-environmental and Biological Sciences, University of Trás-os-Montes and Alto Douro, Vila Real, Portugal; 5 Newborn Screening, Metabolism and Genetics Unit, Department of Human Genetics, National Health Institute Dr. Ricardo Jorge, Porto, Portugal; 6 Biology Department, Faculty of Sciences, University of Porto, Porto, Portugal; 7 i3S – Health Research and Innovation Institute, University of Porto, Porto, Portugal; 8 Department of Osteology and Biomechanics, University Medical Center Hamburg–Eppendorf, Hamburg, Germany; 9 International Center for Lysosomal Disorders, University Medical Center Hamburg-Eppendorf, Hamburg, Germany; 10 Microbiology and Immunology Lab, Center for Interdisciplinary Research in Animal Health, Faculty of Veterinary Medicine, University of Lisbon, Lisbon, Portugal

**Keywords:** N-acetylglucosamine-1-phosphotransferase, Mucolipidosis type II alpha/beta, Antisense oligonucleotides, *GNPTAB*, Exon-skipping RNA therapies

## Abstract

**Background:**

Mucolipidosis type II (ML II) is a lysosomal storage disorder caused by deficiency of N-acetylglucosamine-1-phosphotransferase (GlcNAc-PT), which impairs the trafficking of lysosomal hydrolases. Of all ML II pathogenic variants, c.3503_3504del in *GNPTAB* exon 19 is the most prevalent, therefore constituting a compelling molecular target for the development of personalized therapeutic strategies. Here, we explored the feasibility of an innovative RNA based-therapeutic strategy using antisense oligonucleotides (ASOs) designed to induce exon 19 skipping in *GNPTAB* pre-mRNA. This approach was previously successfully tested at the mRNA level in fibroblasts from ML II patients, where it generated an in-frame mRNA. In the present study, our aim was to evaluate whether *GNPTAB* exon 19 skipping could increase GlcNAc-PT levels and consequently improve the cellular phenotype of ML II patients carrying this pathogenic variant. To address this, we designed a functional approach based on the overexpression of a *GNPTAB* construct carrying the exon 19 deletion enabling the indirect evaluation of the resulting protein´s functionality.

**Results:**

Our first results demonstrated that in ML II fibroblasts, ASO treatment led to a modest increase in lysosomal hydrolase activity at 24 h and 48 h. Moreover, LAMP-1 expression remained elevated and comparable to untreated ML II cells, indicating that GlcNAc-PT activity was not restored. To further investigate the functional relevance of exon 19 skipping, overexpression studies were performed in HEK293T cells. Three constructs (pGNPTAB WT, pGNPTAB del_exon19 and pGNPTAB c.3503_3504del) were expressed. Both pGNPTAB WT and pGNPTAB del_exon19 constructs produced the α/β-precursor. However, only the WT construct generated the mature β-subunit, whereas the pGNPTAB c.3503_3504del construct showed no detectable expression. These findings indicate that exon 19 is essential for proper GlcNAc-PT processing and enzymatic activity.

**Conclusions:**

Although ASO treatment corrected splicing at the mRNA level, it did not restore GlcNAc-PT activity in ML II patient cells. Nonetheless, our findings clarify the functional importance of exon 19 and demonstrate that overexpression of an exon-skipped construct provides a simple and effective strategy to indirectly assess protein functionality, supporting the prioritization of this kind of approach to test an exon-skipping ASO-based approach before advancing to studies in patient-derived cells.

**Supplementary Information:**

The online version contains supplementary material available at https://doi.org/10.1186/s13023-026-04430-2.

## Background

Mucolipidosis type II (ML II, OMIM 252500, ORPHA 576) is a monogenic autosomal recessive lysosomal storage disorder (LSD), with an estimated combined incidence ranging from 0.22 to 2.70 per 100,000 live births (reviewed in [1]). Clinical manifestations typically appear shortly after birth and include severe psychomotor developmental delay, skeletal and cartilage abnormalities, gingival hypertrophy, and cardiopulmonary complications. The skeletal system is particularly affected, with progressive bone deformities and growth impairment. The disease follows a rapidly progressive course, and most patients do not survive beyond the first decade of life [2].

In contrast to most LSDs, ML II is not caused by a deficiency in a single lysosomal enzyme. Instead, it results from pathogenic variants in the enzyme N-acetylglucosamine-1-phosphotransferase (GlcNAc-PT), which is anchored to the *cis*-Golgi membrane by transmembrane helices (TM). This enzyme complex performs a crucial role in tagging approximately 70 different soluble hydrolases with mannose 6-phosphate (M6P) residues. Without M6P residues, the hydrolases fail to be recognized by M6P receptors and are missorted outside the cell instead of being targeted to the lysosome, where they are essential for macromolecule degradation [3, 4].

**Fig. 1:**
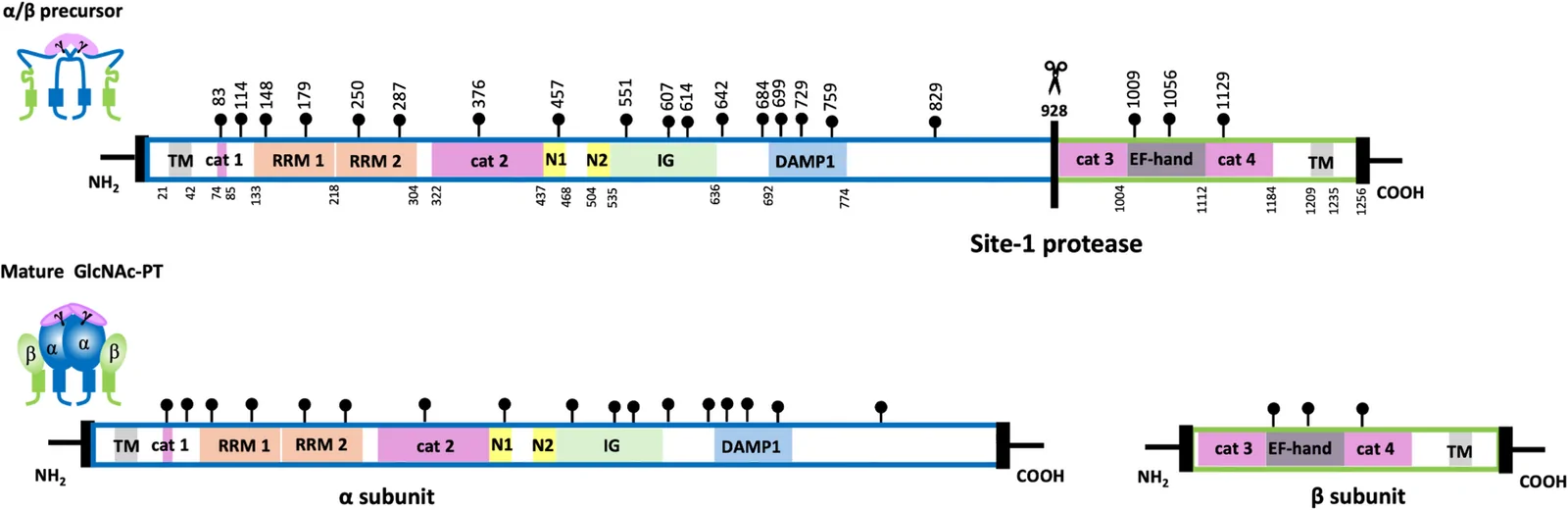
Representation of the GNPTAB protein domains, including glycosylation sites (bold black circle with a vertical line). The α- and β-subunits of GNPTAB share the transmembrane helices (TM) and a conserved Stealth domain (cat 1–cat 4), which together form the enzyme’s catalytic core. In the α-subunit, additional domains are present, including two RNA recognition motif-like regions (RRM1 and RRM2) with still unclear roles, as well as two Notch-like domains (N1 and N2), an immunoglobulin-like module (IG), and a DNA methyltransferase associated protein (DMAP1) domain, all thought to contribute to the specific recognition of lysosomal hydrolases. The β-subunit, in turn, carries an EF-hand domain, which is believed to support the structural stability of the complex. (Adapted from [6])

GlcNAc-PT is an ~ 400-kDa heterohexameric enzyme composed of three distinct subunits: α_2_, β_2_, and γ_2_. The membrane-bound α- and β-subunits are derived from a single precursor protein encoded by the *GNPTAB* gene and synthesized in the endoplasmic reticulum (ER). The soluble γ-subunits are encoded by the *GNPTG* gene. After trafficking from the ER to the *cis*-Golgi apparatus, the catalytically inactive α/β-precursor of GlcNAc-PT undergoes proteolytic processing by the Golgi-resident site-1 protease (S1P). This cleavage, occurring between Lys928 and Asp929, gives rise to the mature, enzymatically active α- and β-subunits [3, 5] (Fig. 1).

Both α- and β-subunits contain multiple domains, including the Stealth domain, which comprises four conserved segments (cat 1 to cat 4) distributed along the α/β-polypeptide chain, which form the catalytic core of the enzyme. In addition to the catalytic region, the α-subunit contains two RNA recognition motif–like domains, two Notch-like domains, an immunoglobulin-like domain, and a DMAP1 domain. The β-subunit contains an EF-hand domain [6–8] (Fig. 1).

The most frequent pathogenic variant in patients with ML II is the frameshift variant c.3503_3504del (p.Leu1168Glnfs*5) in the exon 19 of *GNPTAB* gene [4, 9]. This dinucleotide deletion origins a premature stop codon, leading to a truncated α/β-precursor protein that lacks the C-terminal transmembrane domain and cytosolic sorting motif required for ER to Golgi transport [2, 10, 11].

Currently, there is no approved treatment for ML II. The complexity of the transmembrane GlcNAc-PT, and the fact that the enzyme complex is encoded by two different genes, makes therapeutic options for this severe disease extremely limited.

**Fig. 2:**
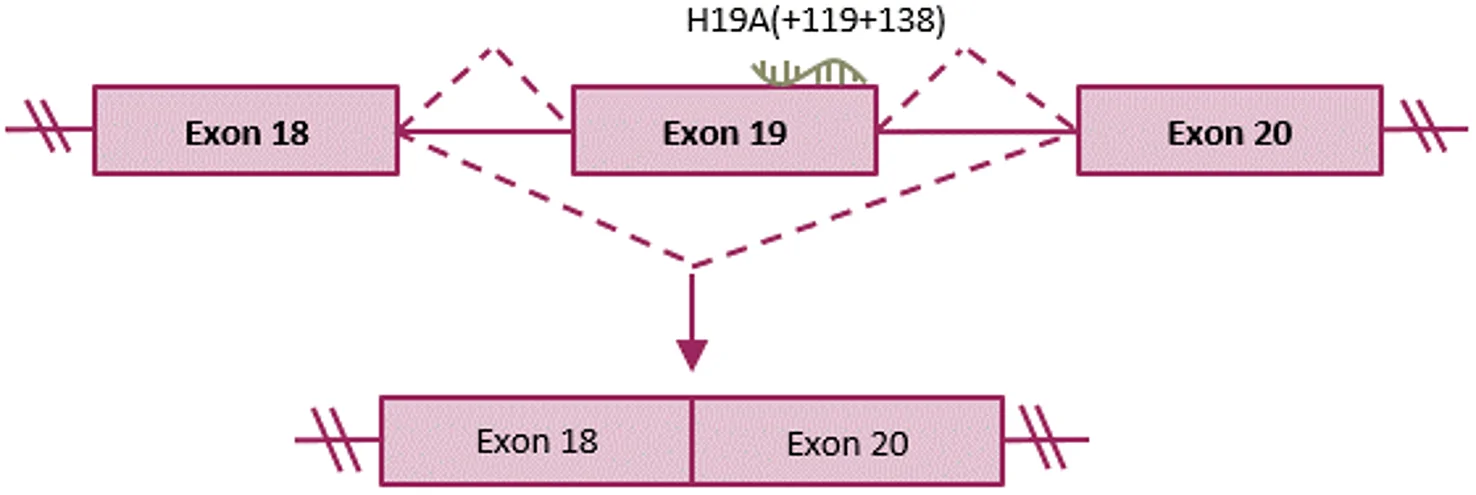
Schematic representation of the binding target region of the 2’-O-Methyl ASO H19A(+119+138) on *GNPTAB* exon 19, along with the splicing pattern that results from its action. From the four SSOs tested in the previous study [9], only the ASO tested in the present work is depicted. Exons 18 and 20 as well as all the involved flanking intronic regions are also represented

In this study, we aimed to advance the development of a therapy for ML II patients carrying the c.3503_3504del pathogenic variant, which due to its high prevalence worldwide is therefore relevant for a considerable number of patients. This variant shows a remarkably wide geographical distribution, being the most common ML II pathogenic variant globally, accounting for 45% of mutant alleles in Portugal and Brazil, 51% in Italy, and 50% among the middle east regions community, respectively [11–14]. Our strategy targets the disease at the RNA level using a specific type of RNA-based therapy to induce the skipping of exon 19, where the pathogenic variant occurs. It employs antisense oligonucleotides (ASOs), short single-stranded molecules of approximately 20 nucleotides that bind with high specificity to target RNA sequences via Watson–Crick base pairing. Specifically, splice-switching oligonucleotides (SSOs), a subclass of ASOs, were used by our team to modulate pre-mRNA splicing and induce *GNPTAB* exon 19 skipping, restoring the reading frame disrupted by the variant (Fig. 2). This method was previously shown to be successful at the RNA level [9]. Our approach is further supported by the clinical approval of several SSO therapies for Duchenne muscular dystrophy, which are based on the same exon-skipping mechanism of action [12, 13].

This study evaluated whether exon 19 skipping can produce a functional GlcNAc-PT enzyme, despite the loss of 56 amino acids, and restore M6P-dependent lysosomal targeting, thereby improving the ML II cellular phenotype. We first performed indirect assays, such as measuring M6P-dependent lysosomal hydrolase activities, after SSO transfection, but these did not conclusively demonstrate therapeutic benefit. We then used an established overexpression system in HEK293T cells to compare wild-type *GNPTAB* with exon 19–deleted and c.3503_3504del constructs. Although the construct without the exon 19 generated the precursor protein, it did not produce the active β-subunit, demonstrating that exon 19 skipping does not restore GlcNAc-PT activity. These results highlight overexpression models as a straightforward way to assess exon-skipping strategies before SSO testing.

In summary, assessing the ASO therapeutic potential typically requires multiple steps, including the selection of an optimal target region, precise ASO design, and validation of splicing efficiency both at the mRNA and protein levels. These stages demand extensive laboratory work and considerable resources, while giving no guarantees of definitely validating our working hypothesis, especially if no direct readout assays are available. That was indeed the case with this study, in which this traditional approach did not allow to obtain an answer regarding the GlcNAc-PT recovery after the SSO approach. Thus, to overcome this issue, we propose an alternative strategy based on the overexpression of a construct carrying the desired exon-skipping. This approach provides a direct means of testing protein functionality and could be particularly advantageous for complex enzymes such as GlcNAc-PT.

## Methods

### Cell culture

Primary skin fibroblasts were obtained from a patient with ML II, carrying the homozygous pathogenic frameshift variant in the *GNPTAB* gene (c.3503_3504del). These cells were provided by the Cell Line and DNA Biobank from Patients Affected by Genetic Diseases (Istituto Giannina Gaslini, Genoa, Italy), which is part of the Telethon Network of Genetic Biobanks. The confidentiality of personal data was respected at all times. Human adult dermal fibroblasts (HDFa) were purchased from Thermo Fisher Scientific (Waltham, MA, USA) and used as a control in all experiments. The HEK293T cell line was kindly provided by Prof. John Neidhardt from Oldenburg University, Germany.

All the cells were cultured following standard conditions in Dulbecco’s Modified Eagle’s Medium (DMEM; Gibco™, Life Technologies, Carlsbad, CA, USA), supplemented with 10% fetal bovine serum, 1% penicillin/streptomycin antibiotic, and 1% amphotericin B antimicotic (all from Gibco™, Thermo Fisher Scientific, Waltham, MA, USA). Cultures were maintained in an incubator at 37 °C, with a humidified atmosphere containing 5% CO_2_.

### Antisense oligonucleotide targeting *GNPTAB* exon 19

The ASO used in the experiments of the present study was a 2’-O-Methyl (2’-O-Me) ASO, named H19A(+119+138) designed to target a *GNPTAB* exon 19 Exonic Splicing Enhancer (ESE) region, which was selected in our previous study [9], as the most efficient ASO in inducing the *GNPTAB* exon 19 skipping. The H19A(+119+138) ASO has the sequence 5’ CGGUUUCGAUACUCUCUUGG 3’ and was acquired from Eurogentec (Seraing, Belgium).

The scramble 2’-O-Me ASO (5’ CCUAUAGGACUAUCCAGGAA 3’) used as a control was synthesized and purified by IDT (Integrated DNA Technologies, Coralville, Johnson County, IA, USA).

### *GNPTAB* vector constructs

For the expression of the human wild-type *GNPTAB*, the pcDNA4^TM^/TO/hGNPTAB-myc-His construct was used. This construct harbours the total *GNPTAB* cDNA sequence, a *c-myc* epitope tag and a polyhistidine region (p*GNPTAB* WT) [14]. The p*GNPTAB* del_exon19 construct, having the deletion of *GNPTAB* exon 19, and the p*GNPTAB* c.3503_3504del construct with a dinucleotide deletion (TC) in the *GNPTAB* exon 19 were generated by GenScript (Rijswijk, Netherlands) using the pcDNA4^TM^/TO/hGNPTAB-myc-His construct as template. The integrity of all inserted sequences was verified by sequencing in an ABI 3500 Genetic Analyser (Applied Biosystems, Foster City, CA).

### Transfection and RT-PCR analysis

Control and ML II fibroblasts, cultured in 6-well plates to 80–90% confluence, were transfected with 500 nM of the 2’-O-Me ASO H19A(+119+138) using Lipofectamine^®^ 2000 reagent (Invitrogen, Carlsbad, CA, USA), in accordance with the manufacturer’s instructions. Also, an unrelated scramble ASO was transfected in the same conditions to confirm the specificity of the assays. Cells were then harvested at 24 h, 48 h and 72 h post-transfection.

Similarly, HEK293T cells cultured in 6-well plates to 80–90% confluence were transfected with 1 µg of each of the three different constructs: p*GNPTAB* WT, p*GNPTAB* del_exon19, and p*GNPTAB* c.3503_3504del using Lipofectamine^®^ 2000 reagent and harvested 24 h after transfection. In the different experiments, the cell pellets were split for protein and RNA analysis. Total RNA was extracted using the GRS Total RNA Kit – Blood & Cultured Cells (GRiSP Research Solutions, Porto, Portugal), and cDNA synthesis was performed with the Ready-To-Go™ You-Prime First-Strand Beads Kit (Cytiva, Marlborough, Massachusetts, USA), according to the manufacturers’ instructions. The RT-PCR analysis of the transfected control and ML II fibroblasts was performed using the following primers: *GNPTAB* - Exon 17 F (5′-CCAGTAACTGACAAAATCCA-3′) and *GNPTAB* - Exon 21 R (5′-ACAGGTCCATGAGCAAATTC-3′). For the HEK293T cells transfected with the different constructs, the RT-PCR was performed using the primer *GNPTAB* - Exon 17 F (5′-CCAGTAACTGACAAAATCCA-3′) and the vector-specific primer BGH reverse (5′-TAGAAGGCACAGTCGAGG-3′). The amplification program was the same for both cases, consisting of 35 cycles at 94 °C (45 s), 57 °C (45 s), and 72 °C (45 s). RT-PCR products were then analysed on 2% agarose gels and all cDNA fragments obtained were cut off from gel for purification with the Wizard^®^ SV Gel and PCR Clean-Up System (Promega, Madison, WI). Direct DNA sequencing was carried out, and to confirm transcript identity, sequences were compared with the *GNPTAB* reference sequence ENSG00000111670 (www.ensembl.org) using the Clustal Omega bioinformatics tool (https://www.ebi.ac.uk/jdispatcher/msa/clustalo?stype=dna).

### Measurement of lysosomal enzyme activities

To measure lysosomal enzyme activities, control and ML II fibroblast pellets were obtained after ASO transfection as described in *Transfection and RT-PCR analysis* and then homogenized in PBS with 0.1% (v/v) Triton X-100. Each resulting homogenate was sonicated three times at 40% power and 40% amplitude for 6 s. Protein concentration was determined using the Pierce™ BCA Protein Assay Kit (Thermo Fisher Scientific, Waltham, MA, USA). The enzymatic activity of β-glucosidase, α-galactosidase A, β-galactosidase, total hexosaminidase, and β-glucuronidase was then assessed using standard protocols previously described in the literature [15–20].

### Protein analysis

#### Western blotting

For SDS-PAGE and Western blot analysis, the pellets of transfected HEK293T cells or from control and ML II patient fibroblasts harvested after transfection as described in *Transfection and RT-PCR analysis*, were resuspended in a solution of PBS containing 0.2% (v/v) Triton X-100 supplemented with a 1X protease inhibitor cocktail (Sigma-Aldrich, St. Louis, MO, USA). The extracts were kept on ice for 30 min with a resuspension every 10 min, followed by centrifugation at 10 000 x *g* for 15 min at 4 °C. Total protein quantification of the cell lysates was carried out with the Pierce™ BCA Protein Assay Kit.

A total of 30 µg of proteins was loaded on an SDS-PAGE 4–15% Mini-PROTEAN^®^ TGX Stain-Free™ Protein Gel (Bio-Rad Laboratories, Hercules, CA, USA) and separated by electrophoresis. Proteins were then transferred to a nitrocellulose membrane (Whatman^®^, Maidstone, UK) using a 400 mA current for 60 min, in a Mini-PROTEAN^®^ Tetra System (Bio-Rad Laboratories, Hercules, CA, USA). Of note, for the samples for α-galactosidase A detection, the proteins were transferred to a nitrocellulose membrane in the Trans-Blot Turbo Transfer System (Bio-Rad Laboratories, Hercules, CA, USA) following a standard protocol according to manufacturer’s instructions.

The membranes were blocked in 5% non-fat dry milk in TBS containing 0.1% Tween-20 (TBS-T) and incubated overnight at 4 °C with each respective primary antibody (Table 1) diluted in TBS-T, followed by incubation with appropriate HRP-coupled secondary antibody (Table 1) for 2 h at room temperature. Equal protein loading of the gels was verified using different control primary antibodies for housekeeping proteins mentioned in Table 1.

Protein signals on the membrane were detected by enhanced chemiluminescence (ECL) using the Amersham™ ECL Western Blotting Detection Reagent (Cytiva, Marlborough, Massachusetts, USA), and Western blot images were captured using the ChemiDoc™ XRS+ imaging system (Bio-Rad Laboratories, Hercules, CA, USA). Finally, quantification of Western blots was performed by densitometric analysis of the band intensities, using the Gel analysis tool of the ImageJ software [21].

#### Antibodies

The antibodies used in this study are listed in Table 1 below.

**Table 1:** List of antibodies

Primary antibody	Dilution	Supplier	RRID
Mouse monoclonal anti-Myc (9B11)	1:1,000	Cell Signaling Technology	AB_2276
Rabbit polyclonal anti-Calnexin (housekeeping)	1:1,000	Enzo Life Sciences	AB_X53616
Rabbit monoclonal anti-galactosidase alpha antibody	1:5,000	Abcam	AB_168341
Goat monoclonal anti-GAPDH (housekeeping)	1:5,000	Santa Cruz Biotechnology	SC_626853
Mouse monoclonal anti-LAMP-1	1:500	Santa Cruz Biotechnology	SC_20357
Mouse monoclonal anti-Actin (housekeeping)	1:5,000	Santa Cruz Biotechnology	SC-8432

Secondary antibody	Dilution	Supplier	RRID
Goat anti-mouse IgG-HRP	1:5,000	Invitrogen™ Thermo Fisher Scientific	AB_31431
Mouse anti-rabbit IgG-HRP	1:5,000	Santa Cruz Biotechnology	SC_2357
Goat anti-rabbit IgG-HRP	1:5,000	Santa Cruz Biotechnology	SC_2004
Donkey anti-goat IgG-HRP	1:5,000	Santa Cruz Biotechnology	SC_2020

HRP: Horseradish Peroxidase; RRID: Research Resource Identifier

### Statistical analysis

All experimental procedures were performed in triplicate, and data is presented as mean ± standard deviation (SD). Statistical analyses were carried out using GraphPad Prism^®^ software (version 9.5.0; GraphPad Software, La Jolla, CA, USA) for macOS. Comparisons between experimental groups were conducted using an analysis of variance (ANOVA). A *p*-value ≤ 0.05 was considered indicative of statistical significance.

## Results

### Evaluation of M6P-dependent hydrolases following ASO transfection in control and ML II patient fibroblasts

Since direct measurement of GlcNAc-PT activity is technically demanding and often lacks sensitivity [22], the diagnosis of ML II is routinely performed based on an indirect assessment of a panel of selected lysosomal hydrolases, such as α-galactosidase A, β-galactosidase, total hexosaminidase, and β-glucuronidase. As an experimental control, the activity of a specific β-glucosidase: β-glucocerebrosidase, is also measured. This lysosomal enzyme travels from the ER to the Golgi complex and endosomes/lysosomes via LIMP-2 (Lysosomal integral membrane protein-2), following a pathway that is independent of the M6P receptor, making it well suited for this assay [23]. This is the diagnostic approach routinely used in our laboratory, and these were the measurements we performed in control and patient fibroblasts, with and without transfection with the 2’-O-Me ASO H19A(+119+138), in order to indirectly assess the presence or absence of GlcNAc-PT enzymatic activity.

**Fig. 3:**
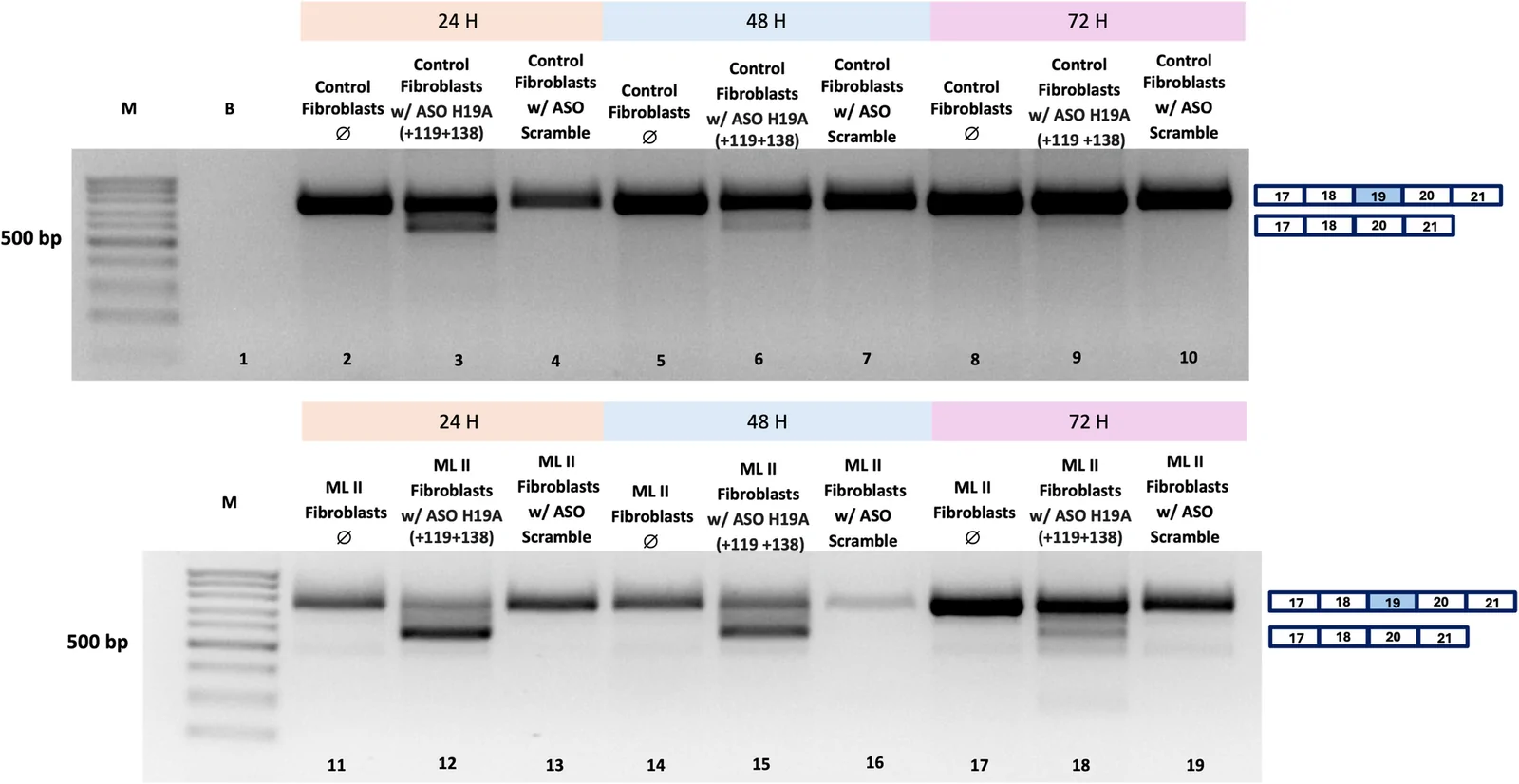
Analysis of *GNPTAB* mRNA fragments (exons 17–21) PCR-amplified from cDNA in control and ML II patient fibroblasts homozygous for thc.3503_3504del pathogenic variant after treatment or not with the 2’-O-Methyl ASO H19A(+119+138) for 24 h, 48 h and 72 h. Control and ML II fibroblasts were also treated with an ASO scramble for 24 h, 48 h and 72 h. On the right, the RT-PCR products are illustrated by schematic drawings. M: molecular weight marker; B: negative control (blank); Control or ML II fibroblasts ∅: sample without ASO; bp – base pairs

In a first step, the presence of transcripts lacking exon 19 after treatment with the ASO H19A(+119+138) was confirmed by RT-PCR analysis. As shown in Fig. 3, the treatment successfully modulated splicing both in control and ML II patient cells. In control fibroblasts, treated with the ASO for 24 h (lane 3) and 48 h (lane 6) it was possible to observe a band corresponding to a transcript with the exon 19 skipping. However, at 72 h post-transfection (lane 9), a visible reduction in band intensity was observed. In ML II patient cells, a similar pattern was observed: a transcript excluding exon 19 was clearly visible at 24 h (lane 12) and 48 h (lane 15), and at 72 h (lane 18) a decrease in band intensity also occurred. The 2’-O-Me ASO scramble, used as negative control, did not affect the *GNPTAB* exon 19 splicing in control (lanes 4, 7 and 10) and ML II fibroblasts (lane 13, 16 and 19), thereby confirming that the ASO H19A(+119+138) was sequence specific.

**Fig. 4:**
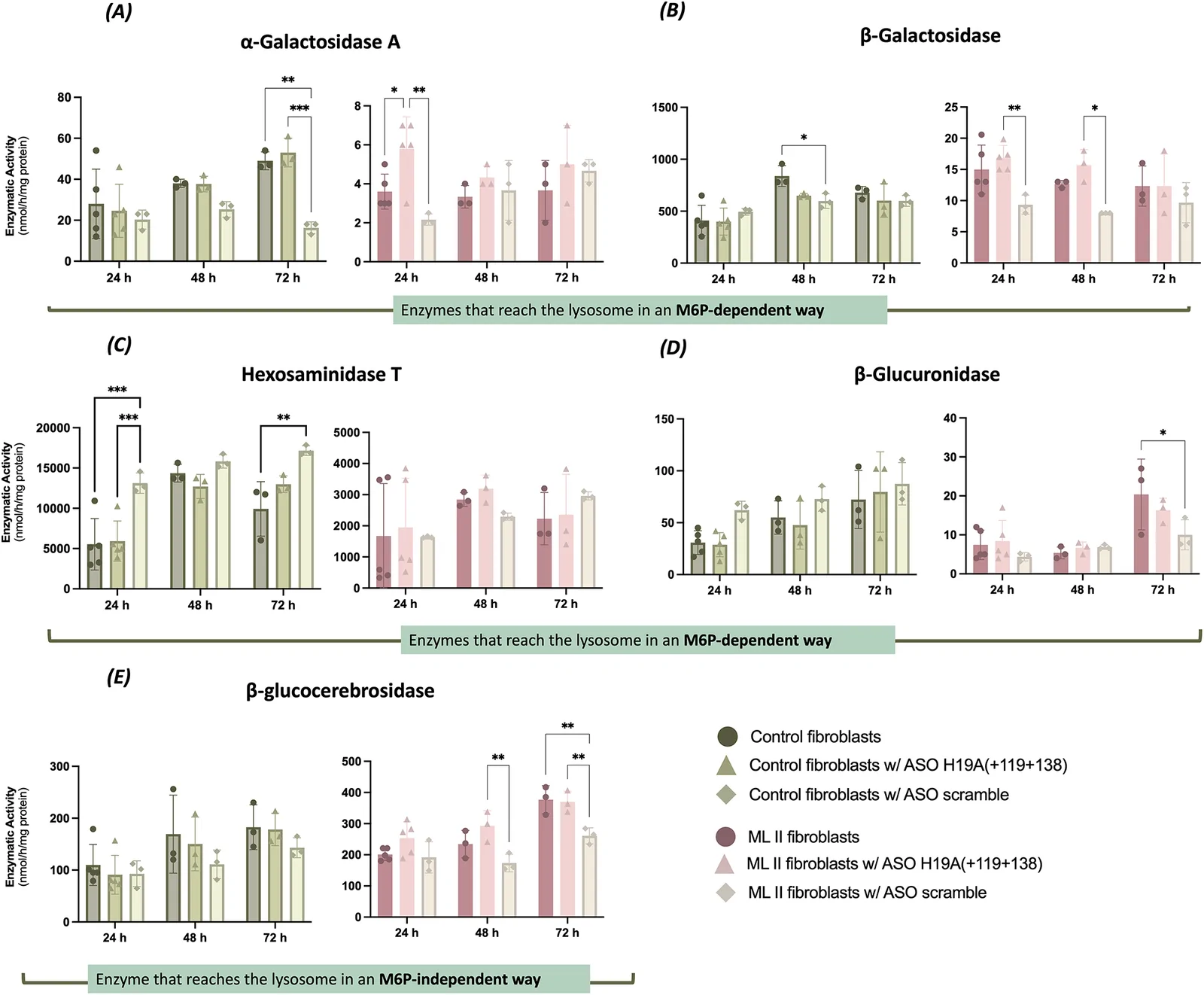
Enzymatic activities of lysosomal hydrolases in control and ML II patient fibroblasts, with and without ASO H19A(+119+138) or ASO scramble transfection were assessed. Graphs were generated using GraphPad Prism 9.5.0 and show the mean ± standard deviation from three independent experiments. Data were analyzed by two-way analysis of variance (ANOVA), followed by Tukey’s multiple comparisons test. * *p* ≤ 0.05, ** *p* ≤ 0.01, *** *p* ≤ 0.001, **** *p* ≤ 0.0001. ∅: sample without ASO; w/: with

In a second step, the same cell samples were used to measure the activities of M6P-dependent hydrolases (Fig. 4A-D). As expected, in untreated ML II patient fibroblasts (depicted in dark pink), the intracellular activities of M6P-dependent hydrolases were significantly lower than the ones observed in to healthy controls (depicted in dark green) at 24 h, 48 h and 72 h. In ML II patient cells treated with the ASO H19A(+119+138) (depicted in light pink), a slight upward trend in enzymatic activity was observed for β-galactosidase (Fig. 4B), total hexosaminidase (Fig. 4C), and β-glucuronidase (Fig. 4D) at 24 h and 48 h. However, these increases were not statistically significant. In contrast, α-galactosidase A activity (Fig. 4A) showed a statistically significant increase in treated ML II patient cells compared to untreated ones 24 h post-transfection. Still, the enzymatic activity levels in ML II treated cells remained well below those observed in normal control. In control fibroblasts (Fig. 4, depicted in light green), treatment with our ASO H19A(+119+138) led to a slight, but not statistically significant decrease in enzymatic activities, as expected. Regarding the M6P-independent enzyme, β-glucocerebrosidase, a slight increase in activity was observed in patient cells, both treated and untreated with our ASO H19A(+119+138) (Fig. 4E). This finding may be explained by a compensatory mechanism, since M6P-dependent enzymes are missorted and fail to reach the lysosome, the resulting substrate accumulation may trigger an upregulation of β-glucocerebrosidase (M6P-independent hydrolase) to mitigate the defect [19].

**Fig. 5:**
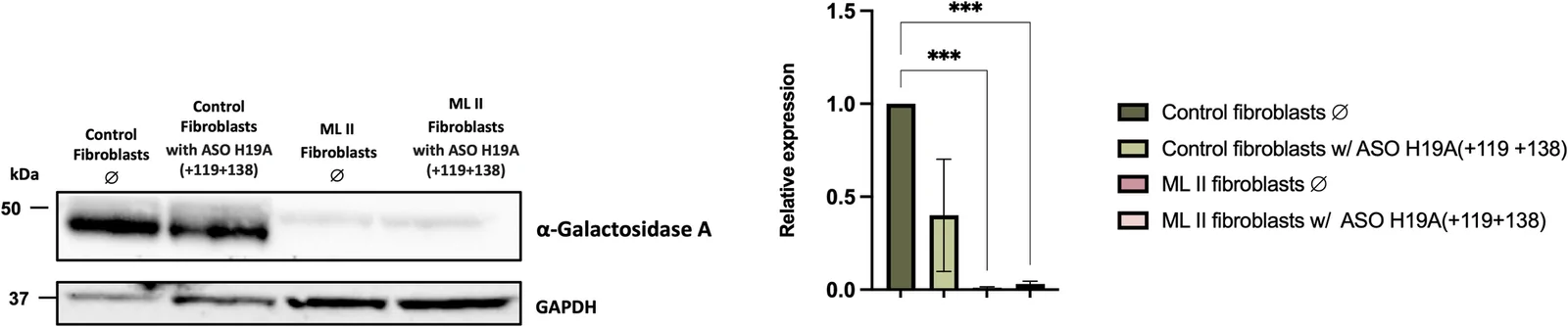
Representative western blot of α-galactosidase A in cellular extracts from control and ML II patient fibroblasts, with and without ASO H19A(+119+138) treatment. Densiometric analysis of α-galactosidase A levels were normalized for GAPDH, which was used for loading gel control, and analysed at ImageJ. The positions of the molecular mass marker proteins (in kDa) are indicated. Three independent experiments were performed. Data were analyzed by one-way analysis of variance (ANOVA). * *p* ≤ 0.05, ** *p* ≤ 0.01, *** *p* ≤ 0.001, **** *p* ≤ 0.0001. ∅: sample without ASO; w/: with

Next, we evaluated α-galactosidase A expression in cell extracts by Western blot analysis. ML II cells, both untreated and treated with our ASO displayed similar protein expression of α-galactosidase A. In contrast, control cells showed a markedly higher expression of α-galactosidase A when compared with both ML II cells untreated and treated with the ASO H19A(+119+138) (Fig. 5). This indicates that, despite the observed increase in enzymatic activity, such improvement was not reflected at the mature protein expression level.

Overall, these results suggest that treatment with the ASO H19A(+119+138) may not be effective in increasing the activity of the M6P-dependent lysosomal hydrolases within the lysosomes.

### Assessment of LAMP-1 expression in WT and ML II patient fibroblasts following ASO transfection

**Fig. 6:**
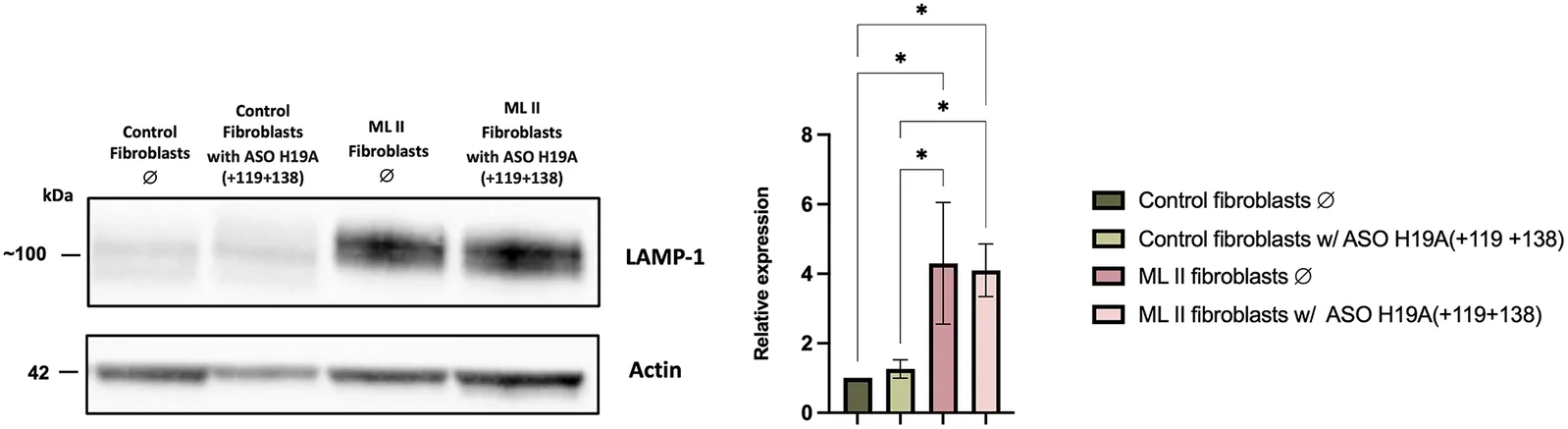
Representative western blot of Lysosomal associated membrane protein 1 (LAMP-1) in cellular extracts from control and ML II patient fibroblasts, with and without ASO H19A(+119+138) treatment. Actin western blot was performed for loading gel control; densiometric analysis of LAMP-1 levels were normalized for actin and analysed at ImageJ. The positions of the molecular mass marker proteins (in kDa) are indicated. Three independent experiments were performed. Data were analyzed by one-way analysis of variance (ANOVA). * *p* ≤ 0.05, ** *p* ≤ 0.01, *** *p* ≤ 0.001, **** *p* ≤ 0.0001. ∅: sample without ASO; w/: with

To determine whether the splicing correction induced by ASO H19A(+119+138) led to changes in key cellular hallmarks of ML II, we analysed LAMP-1 protein expression. LAMP-1, a heavily glycosylated lysosomal membrane protein, constitutes up to 50% of lysosomal membrane proteins and is vital for maintaining lysosomal membrane integrity and function [24]. In ML II cells, LAMP-1 levels are known to be markedly elevated compared to control cells, reflecting the extensive accumulation of undegraded substrates within the lysosomes [25]. Western blot analysis revealed that ML II cells treated with the ASO H19A(+119+138) maintained high LAMP-1 expression levels, closely resembling those of untreated ML II cells. Indeed, a significant difference was observed between control cells and both untreated and ASO-treated ML II cells, indicating that ASO treatment was likely unable to restore GlcNAc-PT activity (Fig. 6).

### Analysis of the α/β-precursor cleavage using overexpression of different vector constructs

**Fig. 7:**
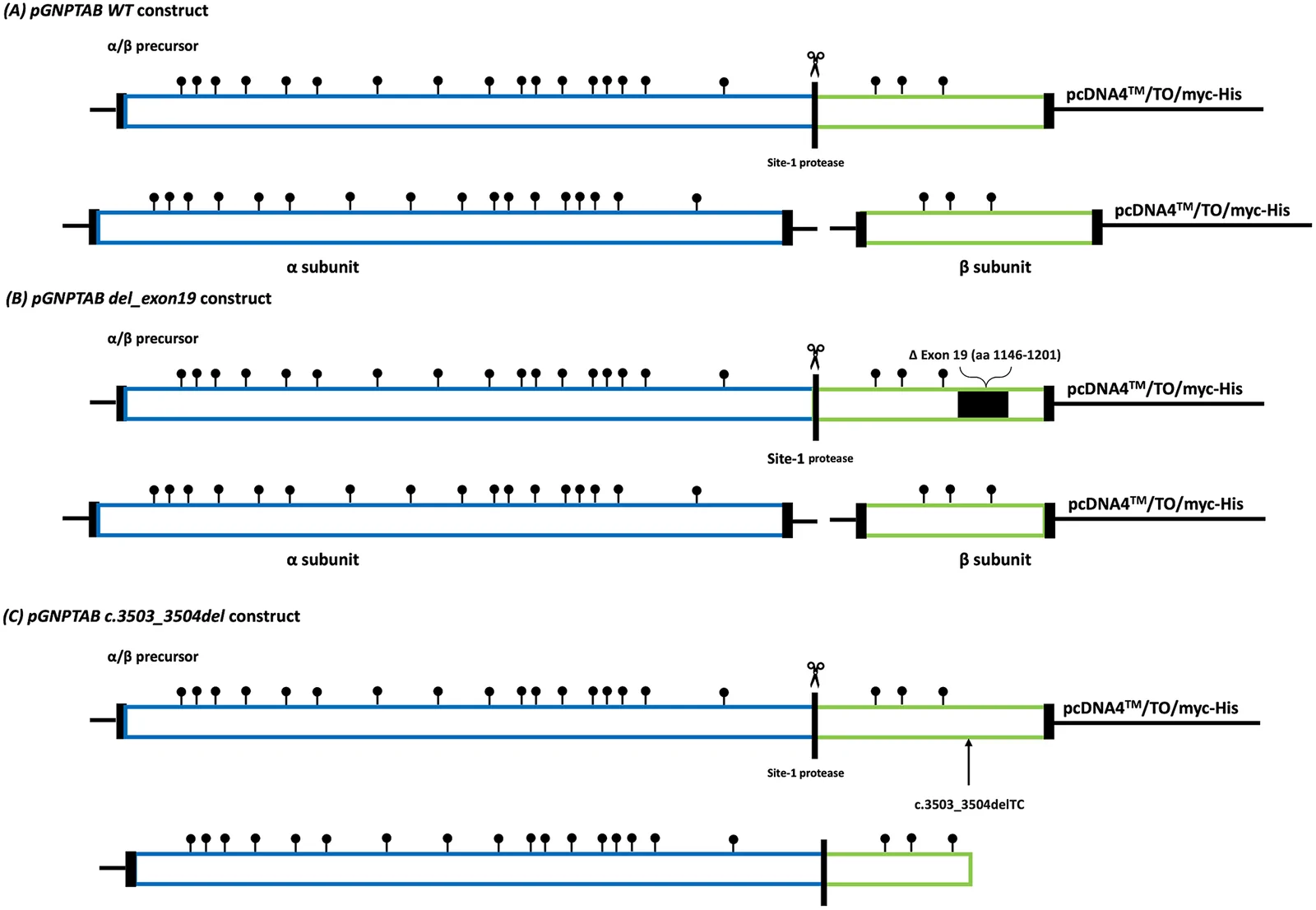
Schematic representation of three different *GNPTAB* constructs generated in the pcDNA4^TM^/TO/myc-His expression vector. (**A**) vector construct with the wild-type *GNPTAB* full-length cDNA sequence (21 exons), (**B**) vector construct with the deletion of exon 19 in the *GNPTAB* cDNA sequence and (**C**) vector construct with the deletion of a dinucleotide TC in exon 19 of the *GNPTAB* cDNA sequence. Each construct scheme has the representation of the specific *GNPTAB* cDNA sequence before and after cleavage by Site-1 protease, including the glycosylation sites (bold black circle with a vertical line)

**Fig. 8:**
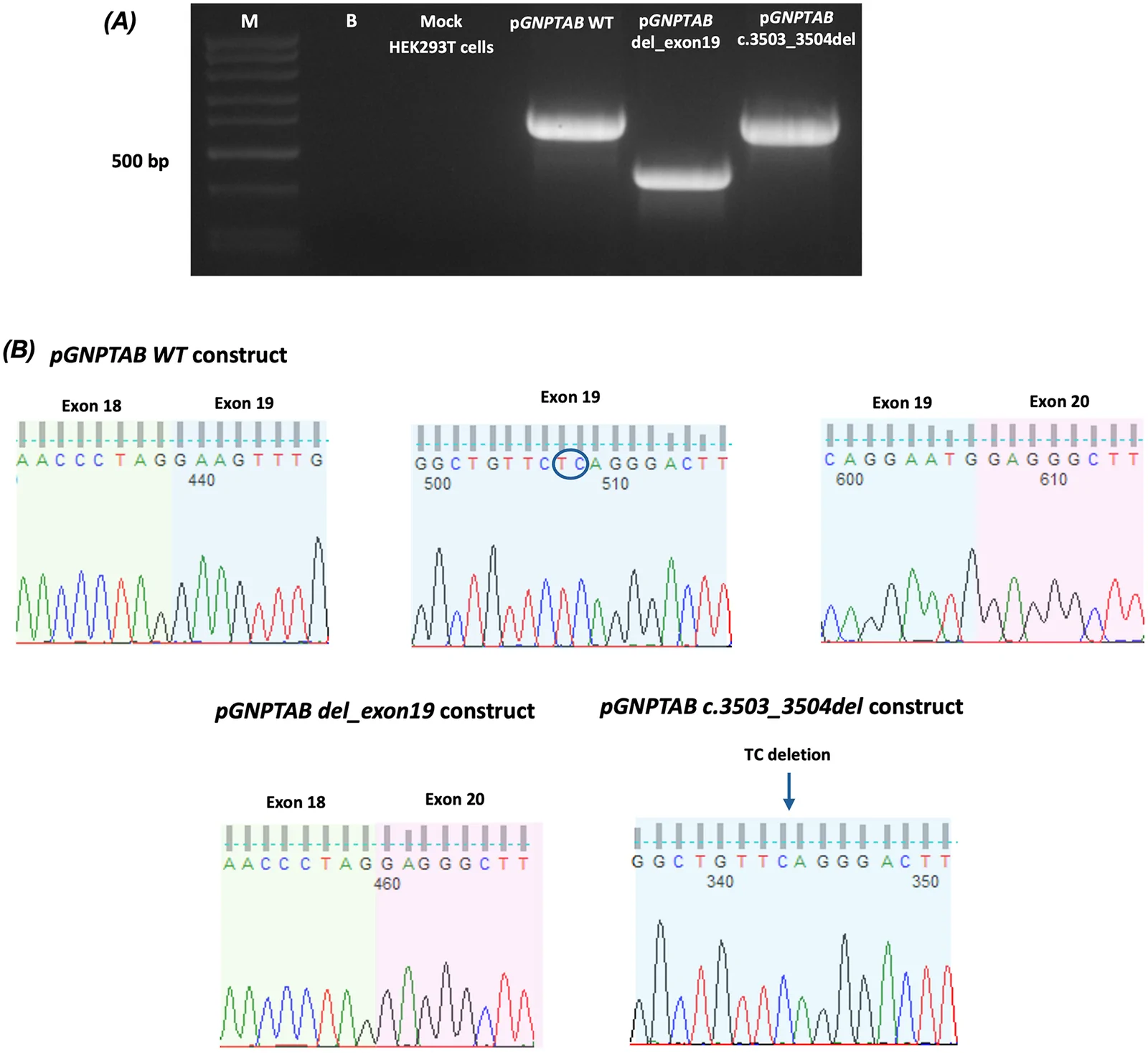
(**A**) Agarose gel analysis of fragments encompassing *GNPTAB* exons 17–21 and part of the vector sequence amplified by PCR from non-transfected HEK293T cells (negative control, Mock), and HEK293T cells transfected with p*GNPTAB* WT, p*GNPTAB* del_ex19 and p*GNPTAB* c.3503_3504del constucts.(**B**) Chromatograms corresponding to Sanger sequencing of p*GNPTAB* WT, p*GNPTAB* del_exon19 and p*GNPTAB* c.3503_3504del constructs. In the p*GNPTAB* WT sequence, the exon 19 TC nucleotides are marked (blue circle), in the p*GNPTAB* c.3503_3504del sequence, the blue arrow indicates the local where the TC deletion occurs in the exon 19. M: molecular weight marker; B: negative control (blank); bp – base pairs

In order to evaluate whether removing exon 19 of *GNPTAB* cDNA sequence to obtain an in-frame transcript could ultimately restore the enzymatic activity of the GlcNAc-PT, we used 3 different constructs: a WT construct (full-length *GNPTAB* cDNA; Fig. 7A), a del_exon19 construct (lacking exon 19; Fig. 7B) and a mutant construct harbouring the c.3503_3504del variant (Fig. 7C). All constructs were generated in the pcDNA4^TM^/TO/myc-His vector, which includes a myc-His⁶ tag (Fig. 7), and were transiently transfected in HEK293T cells. This approach was chosen because to the best of our knowledge, there are no commercially available antibodies able to detect the endogenous levels of GlcNAc-PT [25]. Following transfection, the first step was to verify whether all constructs produced the expected *GNPTAB* expression pattern. PCR analysis using primers targeting the *GNPTAB* exon 17 and the BGH sequence in the vector, together with Sanger sequencing confirmed that each of the 3 different constructs correctly expressed the predicted transcript: p*GNPTAB* WT (619 bp), p*GNPTAB* del_exon19 (451 bp), and p*GNPTAB* c.3503_3504del (617 bp) (Fig. 8).

Next, we examined protein expression and maturation of proteins that are transported from the ER to the Golgi complex. These proteins are initially synthesized as immature precursors on ribosomes bound to the ER, and then guided into the ER lumen by N-terminal signal peptides and subjected to modifications such as N-glycosylation. They are then trafficked to the Golgi apparatus, where glycosylation is further processed. These modifications are highly specific and essential for proper folding, quality control, and subsequent lysosomal targeting [3].

**Fig. 9:**
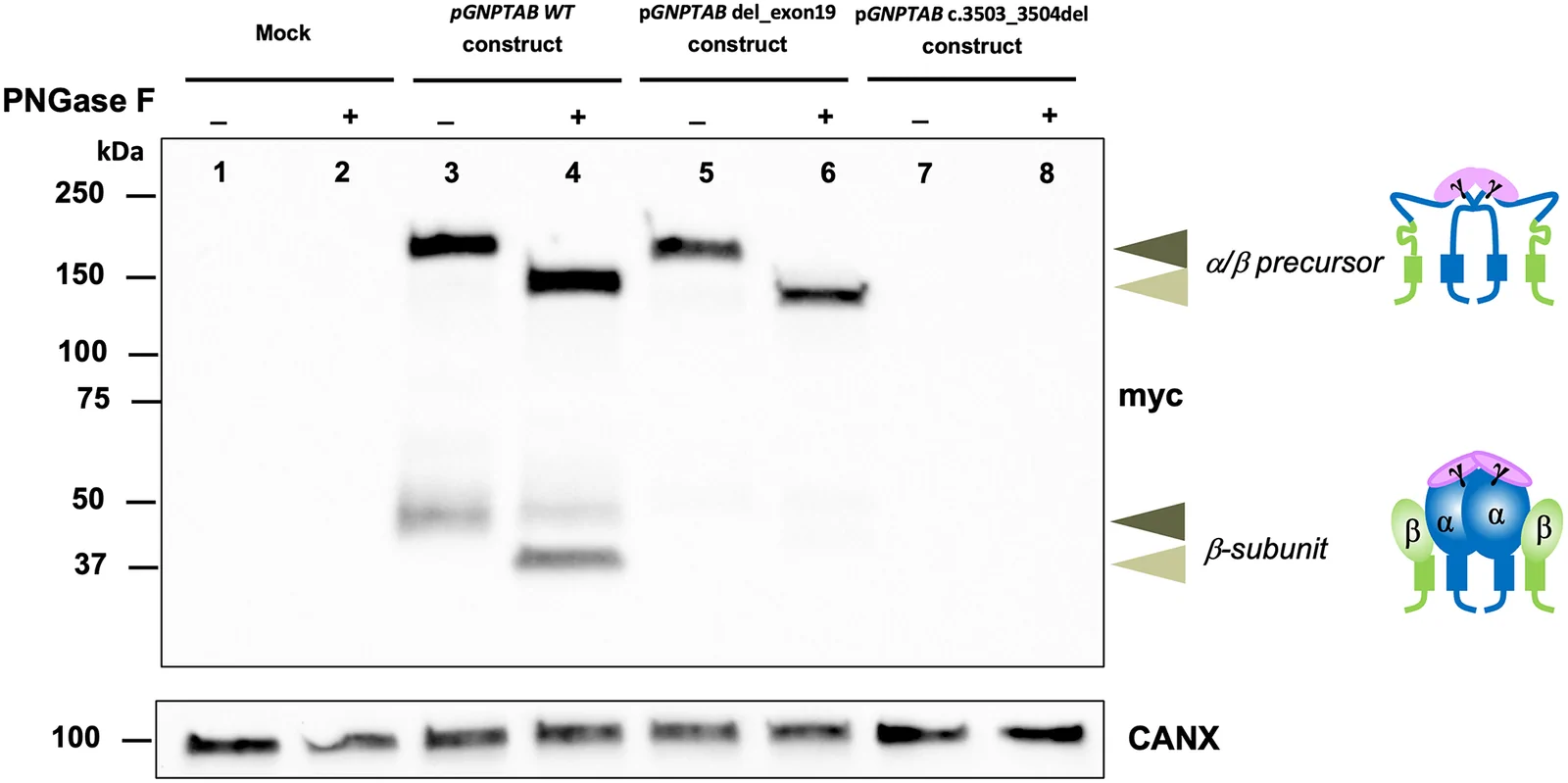
Proteolytic processing of the protein GlcNAc-PT products obtained after the overexpression of the p*GNPTAB* WT, p*GNPTAB* del_exon19 and p*GNPTAB* c.3503_3504del constructs in HEK293T cells. Twenty-four hours after transfection, cell lysates were prepared and incubated for 1 h in the presence (+) or absence (−) of PNGase F, under reducing conditions and then, the western blot analysis was performed using an anti-myc antibody. Lysates from non-transfected cells were used as a negative control. Calnexin (CANX) western blot was carried out for loading gel control. The positions of the protein molecular weight marker (kDa) are indicated. Three independent experiments were performed

To assess whether the GlcNAc-PT α/β-precursor is correctly produced, trafficked through the secretory pathway, and cleaved into α- and β-subunits, several analyses were performed. Due to the C-terminal myc–His sequence present in the GNPTAB precursor protein resulting from the expression of the different constructs, the α/β-precursor and the β-subunit can be detected by a specific anti-myc antibody. After the α/β-precursor cleavage the α-subunit is not detected. However, the primary goal was to verify the presence of the β-subunit, which is shortened by exon 19 removal. Consistently, previous studies using a non-commercial GlcNAc-PT antibody, the only one reported in the literature for this enzyme, recognized and detected specifically only the β-subunit after cleavage [26]. Here, firstly, extracts of HEK293T cells overexpressing p*GNPTAB* WT, p*GNPTAB* del_exon19, and p*GNPTAB* c.3503_3504del constructs were incubated with (+) or without (-) PNGase F, and then, the WB analysis was performed using a myc-tag antibody. PNGase F cleaves all N-linked oligosaccharides that are added to glycoproteins (such as the GlcNAc-PT) during their trafficking from the ER to the Golgi complex. Upon PNGase F treatment, both p*GNPTAB* WT and p*GNPTAB* del_exon19 showed a clear shift in the weight of the α/β-precursor bands (Fig. 9, lanes 4 and 6), indicating that these constructs produce a GlcNAc-PT α/β-precursor that is properly N-glycosylated. As expected, the p*GNPTAB* c.3503_3504del construct showed no expression, with no detectable α/β precursor band (Fig. 9, lanes 7 and 8), consistent with either the degradation of the out-of-frame transcript by nonsense-mediated decay or the degradation of the misfolded protein in the ER. Regarding the expression of the cleaved β-subunit, only the WT construct produced a detectable β-subunit (Fig. 9, lane 3 and 4), suggesting that there is no GlcNAc-PT enzymatic complex assembly activity in the absence of exon 19. Although the α/β-precursor may still be translated, the cleaved β-subunit is not detected (Fig. 9, lane 5 and 6), suggesting that the α/β-precursor without those 56 amino acids is not transported to the Golgi complex or not cleaved by Site-1 protease.

## Discussion

The in vitro validation of an SSO–based therapeutic strategy requires a stepwise evaluation, progressing from confirmation of splicing modulation at the RNA level to assessment of protein expression, processing, and ultimately functional activity. This multi-step validation is particularly critical when targeting complex, multimeric enzymes such as GlcNAc-PT, whose activity depends not only on correct translation but also on precise intracellular trafficking and proteolytic processing.

In a previous study, different 2’-O-Me ASOs were used to alter *GNPTAB* splicing, and successfully induce exon 19 skipping, generating an in-frame transcript lacking the c.3503_3504del pathogenic variant [9].

Our first step was to indirectly assess the effect of the best performing of those ASOs, ASO H19A(+119+138), by measuring lysosomal hydrolases activities. This approach has already been widely used to evaluate GNPTAB-related defects, supporting its validity. For instance, this approach has been applied in studies with ML II fibroblasts [19, 25], ML II iPSCs [19] and *GNPTAB*-knockout DAOY medulloblastoma cells [27]. Similar studies have been reported in *Gnptab* knockout [28] and knock-in mouse models [29], where enzymatic activity was analysed in various tissues, including serum. Together, these findings strengthen the rationale for using lysosomal hydrolases activity as a functional readout to evaluate the impact of our ASO-based strategy. Among the lysosomal hydrolases delivered via the M6P pathway that were analysed, β-galactosidase, β-glucuronidase, and total hexosaminidase showed slight upward trends, although these changes were not statistically significant. In contrast, α-galactosidase A exhibited a statistically significant increase in activity in ML II fibroblasts treated with the ASO, which could suggest minimal residual GlcNAc-PT activity. Nonetheless, the recovered activity remained far below normal levels (Table S1).

Given the slight increase in α-galactosidase A activity observed in enzymatic assays, we decided to analyse this protein by Western blot. However, the analysis revealed an almost complete absence of detectable protein expression in ML II cells treated with the ASO. Importantly, no significant differences were observed between untreated and ASO-treated ML II cells, while a clear difference was evident when these groups were compared with healthy control fibroblasts. Although residual enzyme activity of ~ 10% can be clinically beneficial [30], this is unlikely to be the case here, given the lack of measurable differences between treated and untreated patient cells.

To get further insight into the lysosomal status we examined LAMP-1 expression. The proteins LAMP-1 and LAMP-2 account for nearly 50% of lysosomal membrane proteins, play a crucial role in maintaining lysosomal integrity, and are widely used as standard markers of the lysosome [26]. Consistent with our previous findings, ML II cells treated with the ASO exhibited markedly elevated LAMP-1 levels, comparable to untreated ML II fibroblasts. Similar observations had already been reported in the brain of *Gnptab* knock-in mice, where high LAMP-1 expression was interpreted as reflecting either an increased number or enlarged size of lysosomes, both hallmarks of the disease [29]. Taken together, these results indicate that *GNPTAB* exon 19 skipping was insufficient to restore GlcNAc-PT activity and consequently failed to re-establish proper lysosomal hydrolase delivery.

However, despite these findings, a degree of uncertainty persisted. Indeed, all experiments were carried out in patient-derived fibroblasts, whose slow proliferation rendered the assays required to assess protein correction both time-consuming and costly. These limitations were further compounded by lack of reliable commercial antibodies against the α- and β-subunits of the GlcNAc-PT. To our knowledge, only a single polyclonal antibody targeting the β-subunit has been described, and it is not commercially available [26]. To address the absence of an assay able to provide a direct readout of enzyme activity, or even its presence, we implemented a complementary vector construct-based strategy. Considering the complexity of GlcNAc-PT and the technical limitations to evaluate at the protein level our ASO approach, this alternative strategy provided a direct means of testing whether exon 19 skipping can yield a functional protein. By overexpressing the different constructs in HEK293T cells, we obtained clear evidence that *GNPTAB* exon 19 skipping does not lead to a functional or partially functional GlcNAc-PT protein. The p*GNPTAB* WT construct generated both the α/β-precursor and the cleaved β-subunit indicating correct protein synthesis, trafficking, and processing. In contrast, the p*GNPTAB* c.3503_3504del construct did not produce any detectable protein. This is most likely due to nonsense-mediated mRNA decay, as expected. Alternatively, if translation occurs, this pathogenic variant introduces a premature stop codon, preventing synthesis of the full-length protein [10, 11]. In contrast, the p*GNPTAB* del_exon19 construct produce the α/β-precursor but no cleaved β-subunit was detected. In our previous work we carried out a meticulous in silico analysis to evaluate the potential consequences of removing exon 19, which predicted only the partial loss of the Stealth domain [9]. By then, our assumption that a GlcNAc-PT partially missing that domain would hold potential to function, was mostly grounded on the scarcity of disease-causing pathogenic variants within the missing 56-amino acid stretch. There are four Stealth domains, two located in the α-subunit (cat 1 and cat 2) and two within the β-subunit (cat 3 and cat 4), which seems to play a central role in the catalytic activity of the enzyme [3, 6]. Previous studies have shown that pathogenic variants within these regions can severely impair the enzymatic activity of GlcNAc-PT due to impaired ER-Golgi transport and/or proteolytic activation [31]. Indeed, different pathogenic variants affecting the conserved cat 1 (p.Trp81Leu, p.Asp76Gly and p.Ser385Leu) [11, 32], cat 2 (p.Ser399Phe, p.Glu389Lys and p.Asp408Asn) [11, 33] or cat 3 (p.Arg986Cys and p.His956Tyr) domains [33], have been reported, some of which associated with severe phenotypes and complete loss of enzyme activity (Figure S1) [33]. To further investigate the degree of evolutionary conservation, 40 members of the phosphotransferase family from yeast and bacteria were analysed. This comparison revealed that, among the four catalytic domains, cat 4, the one that comprehends the amino acidic stretch lost as a result of skipping exon 19, is the least conserved [33]. Despite the lower conservation of this region, its absence appears to be critical, as not even partial recovery of GlcNAc-PT enzymatic activity was observed when the exon 19–encoded region was removed. A possible hypothesis is that the lack of exon 19 may impair the correct trafficking of GlcNAc-PT to the Golgi complex, prevent the β-subunit formation, compromise dimer assembly and result in loss of GlcNAc-PT activity. Additionally, previous studies have demonstrated that pathogenic variants affecting structurally important regions of GlcNAc-PT, such as the EF-hand domain, disrupt proper protein folding and lead to retention in the ER rather than correct localization to the Golgi complex. As the EF-hand domain and the cat 3–cat 4 regions contribute to dimer formation, which is required for proteolytic processing and enzymatic activity, structural perturbations are likely to have profound functional consequences [34, 35].

Although it is well established that GlcNAc-PT must be correctly localized to the Golgi complex and undergo S1P-mediated cleavage in order to become active, our results clearly demonstrate that cleavage of the α/β-precursor likely does not occur when the p*GNPTAB* del_exon19 construct is expressed in HEK293T cells.

Overall, while our strategy did not yield the expected therapeutic benefits, these findings hold substantial value by demonstrating that overexpression of engineered constructs serves as an effective, straightforward method for evaluating the functional impact of exon-skipping. This strategy offers a powerful preliminary tool to evaluate SSO-based therapies for rare diseases, before proceeding forward to more complex experiments in patient-derived cells.

## Conclusions

A critical step in the development of ASOs therapies is the establishment of efficient and reliable strategies to evaluate their functional impact. Typically, assessing the therapeutic potential of ASOs involves multiple steps, including careful selection of the target region, precise ASO design, and validation of splicing correction at both the mRNA and protein levels. These processes are laborious, time-consuming, and resource-intensive, particularly when targeting complex enzymes such as GlcNAc-PT. By employing an overexpression system with a construct engineered to carry out the intended exon-skipping event, which can be expressed in commercial cell lines, it is possible to bypass the need for patient cells or other complex disease models. This approach allows for direct evaluation of whether the resulting protein is correctly synthesized and functionally active. Consequently, this strategy provides a rapid and reliable alternative to more indirect or resource-heavy assays, enabling researchers to prioritize the most promising ASO candidates before undertaking extensive studies in patient-derived cells.

## Supplementary Information

Below is the link to the electronic supplementary material.


Supplementary Material 1



Supplementary Material 2


## Data Availability

The original contributions presented in this study are included in the article/Supplementary Materials. Further inquiries can be directed to the corresponding author(s).

## Abbreviations

ML II: Mucolipidosis type II alpha/beta
LSD: Lysosomal Storage Disorder
GlcNAc-PT: N-acetylglucosamine-1-phosphotransferase
TM: Transmembrane helices
M6P: Mannose-6-phosphate
ER: Endoplasmic reticulum
S1P: Site-1 protease
COOH: C-terminal
ASO: Antisense oligonucleotide
SSOs: Splice-switching oligonucleotides
HDFa: Human adult dermal fibroblasts
2’-O-Me: 2’-O-Methyl
PBS: Phosphate buffered saline
TBS-T: Tris-buffered saline containing 0.1% Tween-20
ECL: Enhanced chemiluminescence
WB: Western Blot
HRP: Horseradish Peroxidase
RRID: Research Resource Identifier
SD: Standard deviation
LIMP-2: Lysosomal integral membrane protein-2
GlcNAc: N-acetylglucosamine
CANX: Calnexin
LAMP-1: Lysosomal associated membrane protein 1
