# Supplementary material for: Relevance of functional studies for assessing an antisense oligonucleotide-mediated exon skipping therapeutic strategy for mucolipidosis type II
Source: Orphanet J Rare Dis. 2026 Jun 17;21:309. doi: 10.1186/s13023-026-04430-2 (PMC13528052; doi:10.1186/s13023-026-04430-2)
Supplement: Supplementary file 1 [file 13023_2026_4430_MOESM1_ESM.docx]

**ADDITIONAL FILES**

**Table S1: Lysosomal hydrolase activity ratios in control and ML II fibroblasts after ASO H19A(+119+138) treatment**

| Ratio | | | | | | | | | |
| --- | --- | --- | --- | --- | --- | --- | --- | --- | --- |
|  | ML II |  | ML II |  | ML II |  | ML II w/ ASO |  | Control w/ASO |
|  | Control |  | Control w/ASO |  | ML II w/ ASO |  | Control |  | Control |
|  | α-Galactosidase A | | | | | | | | |
| 24 h | 0,08 |  | 0,10 |  | 0,49 |  | 0,17 |  | 0,83 |
| 48 h | 0,09 |  | 0,09 |  | 0,74 |  | 0,12 |  | 0,99 |
| 72 h | 0,08 |  | 0,07 |  | 0,81 |  | 0,10 |  | 1,08 |
|  | β-Galactosidase | | | | | | | | |
| 24 h | 0,03 |  | 0,03 |  | 0,76 |  | 0,04 |  | 0,96 |
| 48 h | 0,02 |  | 0,02 |  | 0,81 |  | 0,02 |  | 0,77 |
| 72 h | 0,03 |  | 0,03 |  | 1,01 |  | 0,03 |  | 0,89 |
|  | Hexosaminidase T | | | | | | | | |
| 24 h | 0,08 |  | 0,07 |  | 0,55 |  | 0,14 |  | 1,12 |
| 48 h | 0,20 |  | 0,22 |  | 0,87 |  | 0,23 |  | 0,89 |
| 72 h | 0,22 |  | 0,17 |  | 0,94 |  | 0,24 |  | 1,31 |
|  | β-Glucuronidase | | | | | | | | |
| 24 h | 0,24 |  | 0,25 |  | 0,89 |  | 0,27 |  | 0,94 |
| 48 h | 0,10 |  | 0,11 |  | 0,78 |  | 0,12 |  | 0,87 |
| 72 h | 0,28 |  | 0,26 |  | 1,24 |  | 0,23 |  | 1,10 |
|  | β-Glucosidase | | | | | | | | |
| 24 h | 1,68 |  | 2,02 |  | 0,83 |  | 2,03 |  | 0,83 |
| 48 h | 1,38 |  | 1,56 |  | 0,80 |  | 1,73 |  | 0,89 |
| 72 h | 2,06 |  | 2,12 |  | 1,02 |  | 2,03 |  | 0,98 |

Ratio of the enzymatic activities of different lysosomal hydrolases obtained between samples of control and ML II patient fibroblasts with the c.3503_3504del mutation untreated or treated with the ASO H19A(+119+138) for 24 h, 48 h and 72 h. Values represent the mean of three independent experiments.

**Figure S1:** Schematic representation of the GNPTAB protein including the localization of reported pathogenic variants within the catalytic domains. For the cat 1 domain variants, different behaviours were observed: p.Trp81Leu and p.Asp76Gly were shown to lead to retention in the ER, associated with a complete lack of precursor processing [11, 32]; p.Ser385Leu, on the other hand, does not interfere with ER-to-Golgi transport of the α/β-precursor nor with its proteolytic processing, even though the enzyme remains inactive [32]. Regarding the variants identified in cat 2, p.Glu389Lys and p.Asp408Asn result in a complete loss of activity, although the Golgi transport or S1P-mediated cleavage is not affected [33], p.Ser399Phe, on the other hand, while associated with a milder clinical phenotype, is largely retained in the ER and fails to undergo proteolytic processing into the mature α- and β-subunits [11]. Finally, none of the variants identified in cat 3 had their Golgi transport or precursor cleavage affected, indicating that their pathogenicity is mainly due to a marked reduction in GlcNAc-PT activity [33]. The region corresponding to exon 19 skipping is highlighted in black.
