# Supplementary material for: Relevance of functional studies for assessing an antisense oligonucleotide-mediated exon skipping therapeutic strategy for mucolipidosis type II
Source: Orphanet J Rare Dis. 2026 Jun 17;21:309. doi: 10.1186/s13023-026-04430-2 (PMC13528052; doi:10.1186/s13023-026-04430-2)
Supplement: Supplementary file 2 [file 13023_2026_4430_MOESM2_ESM.docx]

**Figure 2**

**Control Fibroblasts**

**ML II Fibroblasts**

**Figure 5**

**α-galactosidase A**

**GAPDH**

**Figure 6**

**LAMP-1**

**Actin**

**Figure 8**

**Figure 8**

**Myc-tag**

**Calnexin**
